# Delayed Presentation of Classic Bladder Exstrophy Associated With Epispadias and Bilateral Non‐Scrotal Testes in an 11‐Year‐Old Afghan Boy: A Case Report

**DOI:** 10.1002/ccr3.73205

**Published:** 2026-07-22

**Authors:** Abdul Ghafar Ghayur, Habibullah Azimi, Murtaza Haidary, Ahmadshah Wazir, Sherin Alem Ibrahimkhail, Khawja Sattar Samim

**Affiliations:** ^1^ Department of Plastic and Burn Surgery Esteqlal Hospital Kabul Afghanistan; ^2^ Medical Research and Technology Center Khatam Al‐Nabieen University Kabul Afghanistan; ^3^ Department of Clinical Laboratory Sciences, Faculty of Medical Laboratory Technology Khatam Al‐Nabieen University Kabul Afghanistan

**Keywords:** Afghanistan, bladder exstrophy, cryptorchidism, delayed presentation, epispadias

## Abstract

Bladder exstrophy–epispadias complex (BEEC) is a rare congenital anomaly due to failure of lower abdominal wall and urinary tract closure. We report an 11‐year‐old boy with neglected bladder exstrophy, epispadias, and bilateral cryptorchidism. He underwent staged reconstruction with successful closure, epispadias repair, and improved postoperative function and quality of life.

## Key Clinical Message

Late presentation of classic bladder exstrophy with epispadias and bilateral non‐scrotal testes continues to pose a major challenge in low‐resource settings. Nevertheless, even in older children, staged reconstructive surgery can provide substantial anatomical restoration, better hygiene, and improved psychosocial outcomes, underscoring the need for timely referral and coordinated multidisciplinary long‐term follow‐up.

## 1 Introduction

Developmental disorders of the genitals represent a heterogeneous group of congenital conditions characterized by atypical development of chromosomal, gonadal, and anatomical sex. These disorders often involve genital structures and may result in long‐term functional and psychosocial consequences [1]. Among them, the bladder exstrophy–epispadias complex (BEEC) is a rare but severe congenital midline defect affecting the urinary bladder, urethra, external genitalia, pelvic floor, and bony pelvis. BEEC comprises a clinical spectrum ranging from isolated epispadias, the mildest form, to classic bladder exstrophy and cloacal exstrophy, the most severe presentation. Epispadias itself is an uncommon anomaly, with an estimated incidence of about one in 101,000 live male births [2], and is characterized by abnormal development of the dorsal urethra and external genitalia due to defective midline closure during embryogenesis [3]. The underlying embryologic mechanism of BEEC is believed to involve abnormal development of the cloacal membrane and impaired mesenchymal migration in early gestation, resulting in failed fusion of the infraumbilical abdominal wall [2]. Cryptorchidism, defined as incomplete descent of one or both testes into the scrotum, is among the most common congenital anomalies in males and is associated with reduced fertility potential and an increased risk of testicular malignancy if left untreated [4].

Management of developmental genital disorders requires a timely multidisciplinary approach to ensure accurate diagnosis, appropriate sex assignment when relevant, optimal timing of surgical interventions, and support for long‐term psychological and social well‐being [5]. Current treatment strategies for bladder exstrophy associated with epispadias and cryptorchidism involve staged surgical reconstruction, including early bladder closure [6], epispadias repair [3], and orchidopexy [7]. These interventions aim to achieve urinary continence, preserve renal function, and improve future reproductive outcomes [2]. Herein, we present a rare case of bladder exstrophy associated with epispadias and cryptorchidism in an 11‐year‐old Afghan boy, emphasizing the clinical features and challenges associated with delayed presentation.

## 2 Case History

**FIGURE 1:**
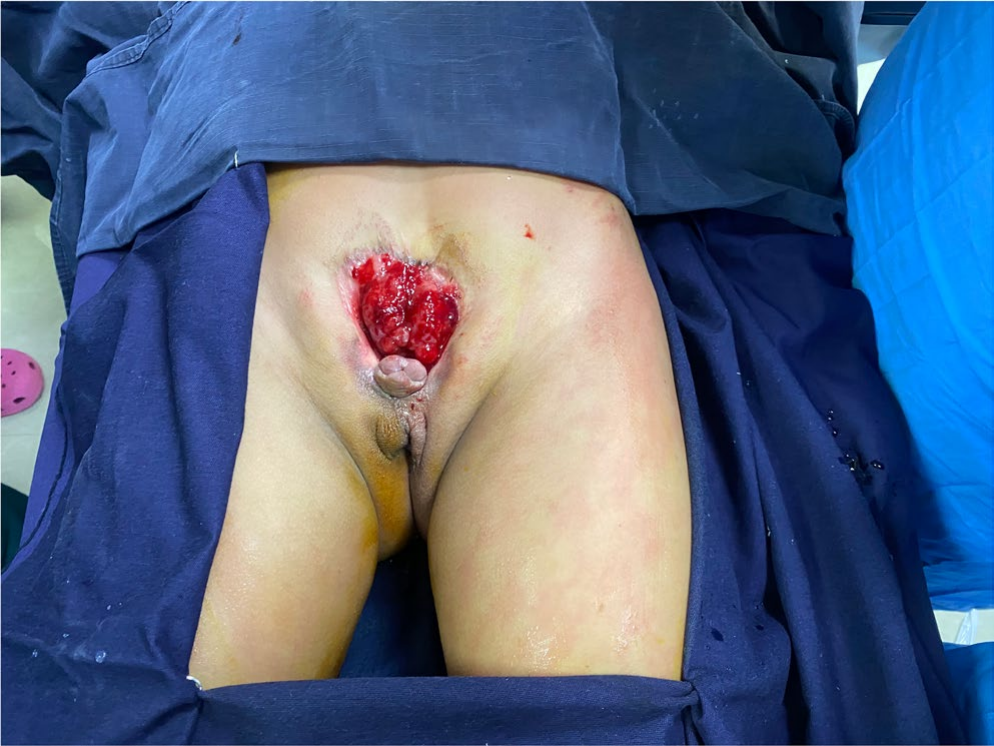
Preoperative clinical photograph demonstrating classic bladder exstrophy with exposed bladder mucosa through a midline infraumbilical abdominal wall defect. Associated complete epispadias with a short penis and underdeveloped empty scrotum, suggestive of bilateral non‐scrotal testes, is also visible.

An 11‐year‐old boy presented to the Plastic and Reconstructive Surgery department with a lifelong history of persistent urinary leakage originating from a defect in the lower abdomen, along with abnormal external genitalia noted since birth. He was born at home through an uncomplicated spontaneous vaginal delivery and had not received any prior surgical assessment or intervention. The delay in seeking medical care was attributed by the family to financial constraints, limited access to specialized healthcare services, and lack of awareness about reconstructive treatment options. There was no known family history of congenital anomalies, and parental consanguinity was not reported. On admission, the patient was alert, cooperative, and hemodynamically stable, with no signs of acute systemic illness. Clinical examination identified a midline infraumbilical abdominal wall defect with exposed bladder mucosa, characteristic of classic bladder exstrophy. Continuous dribbling of urine from the exposed bladder plate was observed. The adjacent skin demonstrated mild irritation consistent with chronic exposure to urine (Figure 1).

## 3 Methods

Examination of the external genitalia revealed a shortened penis with complete epispadias. The scrotum was hypoplastic and bilaterally empty, raising suspicion for undescended testes on both sides. Overall, these findings were consistent with bladder exstrophy accompanied by epispadias and probable bilateral cryptorchidism (Figure 1). Routine laboratory tests—including complete blood count, serum electrolytes, blood urea nitrogen, and creatinine—were within normal ranges. Hormonal analysis showed elevated luteinizing hormone (LH, 9.5 IU/L) and follicle‐stimulating hormone (FSH, 14.9 IU/L), along with reduced serum testosterone (0.8 ng/mL). Karyotyping confirmed a normal male chromosomal pattern (46, XY). Ultrasonography of the urinary tract demonstrated both kidneys in situ without significant hydronephrosis, while the bladder remained externally exposed due to the exstrophy defect. Based on the clinical presentation and imaging findings, a diagnosis of classic bladder exstrophy with complete epispadias and bilateral non‐scrotal testes was made.

**FIGURE 2:**
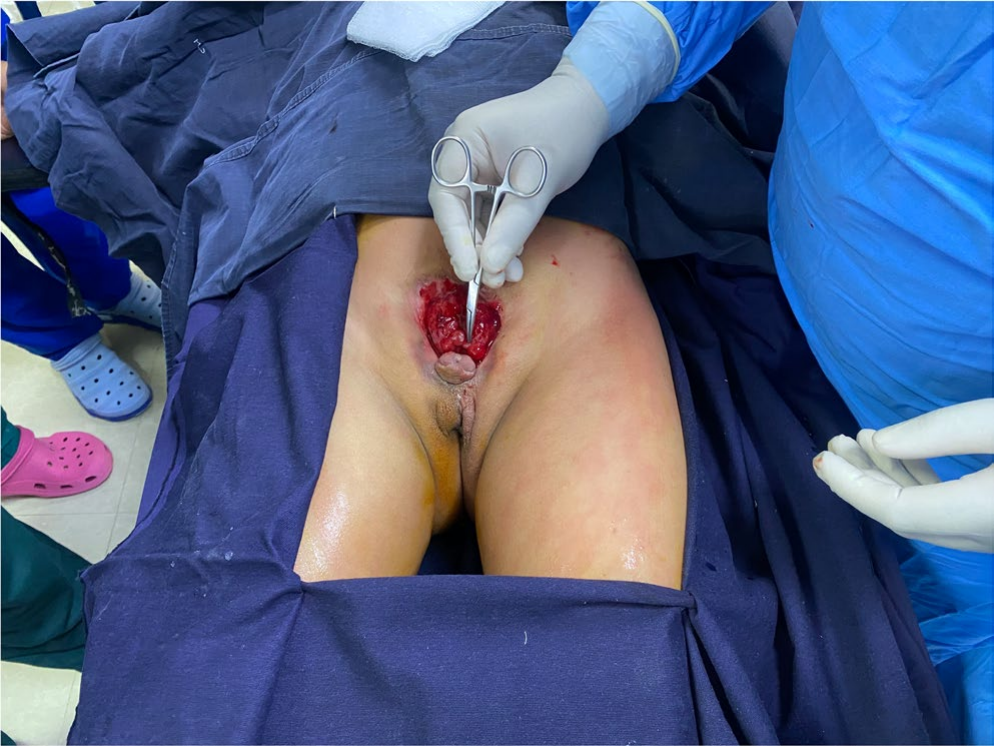
Intraoperative photograph showing mobilization of the exposed bladder plate during first‐stage reconstruction of classic bladder exstrophy. The margins of the exstrophy defect and associated epispadias are visible prior to definitive closure and abdominal wall repair.

**FIGURE 3:**
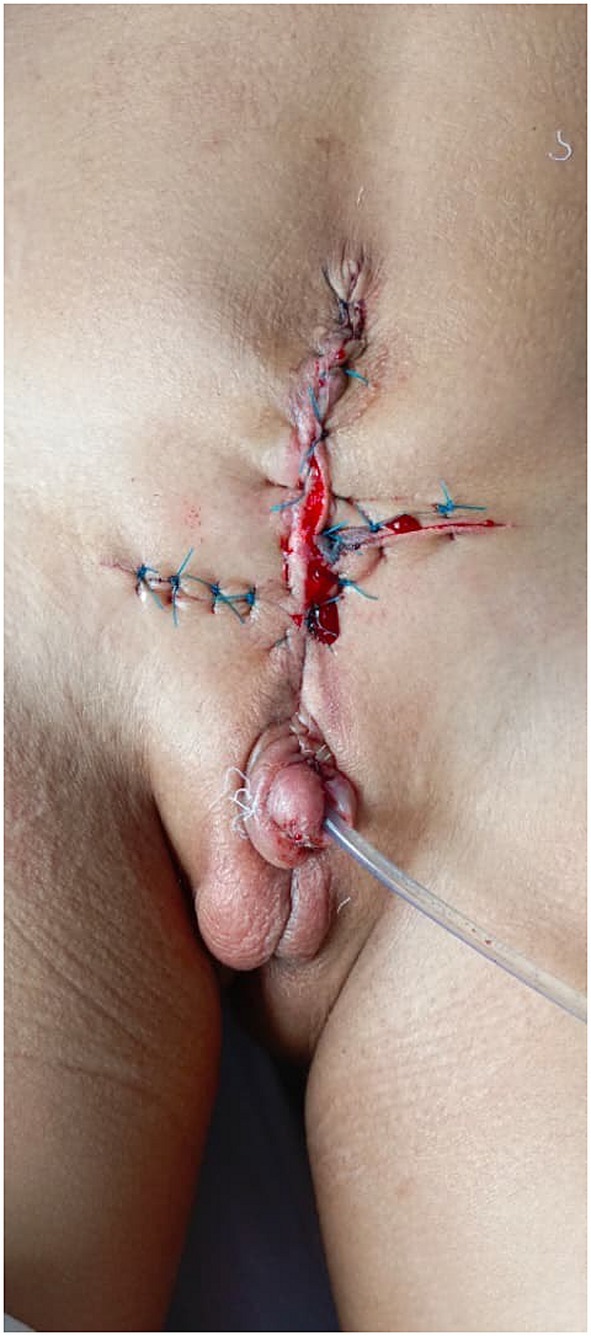
Immediate postoperative photograph demonstrating successful closure of the bladder exstrophy defect and lower abdominal wall reconstruction with intact sutures. Residual epispadias repair has been completed, and urinary drainage is maintained with a urethral catheter.

Following multidisciplinary evaluation and informed consent from the family, a staged reconstructive approach was planned. The first‐stage procedure aimed to achieve closure of the bladder exstrophy, reconstruction of the abdominal wall, and correction of the epispadias. Under general anesthesia, the exposed bladder plate was meticulously dissected from the surrounding tissues and closed in layers. Adequate intraoperative approximation of the pelvic ring was achieved, eliminating the need for pelvic osteotomy (Figures 2 and 3; Video 1). A formal radiographic measurement of pubic symphysis diastasis was not obtained preoperatively. Nevertheless, intraoperative assessment demonstrated satisfactory approximation of the pelvic ring without excessive tension. The lower abdominal wall was then reconstructed through layered soft tissue closure without undue tension. Ureteric catheterization or stenting was not performed. Urinary drainage was maintained with an 8‐Fr urethral Foley catheter. Concurrently, the epispadias was repaired using a modified Cantwell–Ransley technique, involving tubularization of the urethral plate and reconstruction of penile soft tissues (Video 1). Immediate postoperative assessment demonstrated successful closure of the abdominal defect with intact sutures and satisfactory urinary drainage via the catheter (Figure 3). Given the complexity of the primary reconstruction, correction of the bilateral undescended testes was deferred for approximately 3 months, with plans for reassessment and orchidopexy in a subsequent stage. The intended management includes urological evaluation and localization of the testes, followed by staged orchidopexy, with the specific surgical approach determined according to the anatomical position of the testes at reassessment. Following postoperative recovery, the patient was referred to the Urology Department for further evaluation and definitive management of the bilateral non‐scrotal testes.

**VIDEO 1:** Intraoperative video demonstrating progressive surgical dissection and mobilization of the classic bladder exstrophy defect during staged reconstruction. The exposed bladder mucosa and surrounding soft tissues are shown while preparing for layered closure of the bladder plate, lower abdominal wall, and associated genital anomalies. Video content can be viewed at https://onlinelibrary.wiley.com/doi/10.1002/ccr3.73205.

## 4 Outcome and Follow‐Up

The postoperative course was uneventful. The patient remained afebrile, tolerated oral feeding well, and maintained adequate urine output throughout hospitalization. The urethral catheter was removed on postoperative day 14, at which time the surgical wound was clean, dry, and well healed, with no evidence of wound dehiscence, fistula formation, urinary leakage, or surgical site infection. The patient was discharged in stable condition with scheduled outpatient follow‐up. At early follow‐up, the family reported marked improvement in hygiene, physical comfort, and social confidence compared with the preoperative condition, underscoring the substantial physical and psychosocial burden associated with delayed untreated bladder exstrophy in older children. Management of the bilateral non‐scrotal testes remained pending, and staged reassessment with planned orchidopexy was scheduled approximately 3 months after the initial reconstruction. The patient was subsequently referred to the Urology Department for further assessment and definitive management of the bilateral non‐scrotal testes.

## 5 Discussion

BEEC represents a rare spectrum of congenital anomalies arising from failure of normal closure of the lower abdominal wall and urinary tract during embryonic development. Classic bladder exstrophy is typically identified at birth due to the obvious presence of exposed bladder mucosa and associated genital abnormalities [3]. It is usually diagnosed and treated surgically in the neonatal period [8]. Presentation at 11 years of age is therefore highly unusual and highlights ongoing barriers to pediatric surgical care in resource‐limited settings, such as financial constraints, restricted access to healthcare facilities, delayed referral pathways, and limited availability of specialized reconstructive expertise. Only a small number of similar late‐presenting cases in older children and adolescents have been described in the literature [8]. In contrast to neonatal repair, surgical management in older patients is more challenging. Prolonged exposure of the bladder mucosa can lead to inflammation, fibrosis, and reduced bladder capacity, while widening of the pubic symphysis and decreased pelvic flexibility may hinder tension‐free closure. As a result, delayed repair often necessitates more complex reconstructive strategies, including pelvic osteotomy, bladder augmentation, or extended urinary diversion [9].

The association of classic bladder exstrophy with complete epispadias is embryologically expected, as both anomalies result from abnormal development of the cloacal membrane and disrupted mesenchymal migration during early gestation. This leads to failure of fusion of the infraumbilical abdominal wall and urethral structures [3]. Recent molecular research further supports a multifactorial etiology for BEEC, with identified susceptibility loci—such as ISL1—suggesting an interplay between genetic predisposition and environmental influences in its pathogenesis [2].

In males, epispadias has significant clinical implications, as untreated cases can adversely affect urinary continence, penile development, sexual function, and body image. Long‐term outcomes are therefore closely linked to timely and appropriate surgical correction [10]. In this case, performing epispadias repair concurrently with the initial reconstructive procedure was clinically appropriate. Combining genital reconstruction with primary bladder closure in selected bladder exstrophy–epispadias cases can reduce the total number of surgeries and anesthetic exposures while also facilitating earlier psychosocial stabilization when feasible [11].

An additional notable finding in this patient was a bilaterally empty scrotum, suggestive of bilateral cryptorchidism. This condition is more frequently observed in patients with bladder exstrophy than in the general population, largely due to associated developmental abnormalities of the pelvis and lower abdominal wall—such as pubic diastasis and altered pelvic anatomy—which may disrupt normal testicular descent [12]. Deferring orchidopexy in this case was appropriate, as postoperative stabilization following major reconstructive surgery was the immediate priority. However, timely definitive management remains essential, since prolonged cryptorchidism is associated with impaired spermatogenesis, subfertility, increased risk of testicular torsion, and a higher likelihood of testicular malignancy [7].

The abnormal hormonal profile in this patient is clinically significant, as endocrine regulation plays a key role in normal testicular descent. Testicular migration is dependent on intact hypothalamic–pituitary–gonadal axis signaling, particularly adequate LH–mediated stimulation of Leydig cells and sufficient testosterone production [13]. Consequently, alterations in LH, FSH, or testosterone levels may contribute to bilateral undescended testes or impaired descent.

In this case, the observed hormonal disturbances may have served as an additional contributing factor alongside the anatomical abnormalities inherent to bladder exstrophy. This highlights the importance of endocrine evaluation in patients with exstrophy associated with bilateral empty scrotum, as identifying hormonal dysfunction can guide long‐term follow‐up, pubertal surveillance, fertility counseling, and optimal timing of orchidopexy. Furthermore, the presence of a normal 46, XY karyotype is clinically relevant, as it excludes major sex chromosome abnormalities and supports a diagnosis of a structural male developmental disorder affecting the urinary and genital systems, rather than a chromosomal disorder of sex development.

Management of neglected bladder exstrophy in older children is considerably more complex than neonatal repair. Delayed presentation is often associated with an increased requirement for pelvic osteotomy, greater difficulty in achieving tension‐free closure, and higher risks of complications such as fistula formation, wound dehiscence, and urinary tract infection [14]. In addition to anatomical reconstruction, the psychosocial aspect of care is particularly important. Older children and adolescents with bladder exstrophy frequently face psychosocial challenges related to urinary incontinence, repeated hospital visits, and concerns regarding body image and social integration [15].

In this case, the postoperative improvement in comfort and self‐confidence underscores that surgical success should be evaluated not only in terms of continence and renal preservation, but also in relation to psychosocial well‐being and overall quality of life. This case also reflects the ongoing burden of late‐presenting congenital urogenital anomalies in resource‐limited settings such as Afghanistan. Enhancing neonatal screening, establishing effective referral systems, strengthening pediatric surgical services, and improving parental education could significantly reduce preventable long‐term morbidity associated with BEEC. Continued multidisciplinary follow‐up is essential to monitor urinary continence, renal function, pubertal progression, fertility potential, and to complete staged management including planned orchidopexy.

## Author Contributions

**Abdul Ghafar Ghayur:** investigation, data curation. **Habibullah Azimi:** conceptualization, investigation. **Murtaza Haidary:** conceptualization, supervision. **Ahmadshah Wazir:** writing – original draft, data curation. **Sherin Alem Ibrahimkhail:** investigation, validation. **Khawja Sattar Samim:** methodology, writing – original draft, resources.

## Funding

The authors have nothing to report.

## Consent

Written informed consent for publication of this case report and accompanying clinical images was obtained from the patient's legal guardian.

## Conflicts of Interest

The authors declare no conflicts of interest.

## Data Availability

The data that support the findings of this study are available on request from the corresponding author. The data are not publicly available due to privacy or ethical restrictions.
